# The impact of farmers’ participation in green cooperative production on green performance—A study based on the moderating effect of environmental regulation

**DOI:** 10.1038/s41598-024-67167-7

**Published:** 2024-07-20

**Authors:** Zhenghua Zhang, Xiahui Wang, Xingchen Yi, Lun Hu

**Affiliations:** 1 School of Economics and Management, Jiangxi Agricultural University, Nanchang, 330045 China; 2 Research Centre on Finance and Accounting, Jiangxi Agricultural University, Nanchang, China; 3 Rural Revitalization Strategy Research Institute, Jiangxi Agricultural University, Nanchang, China

**Keywords:** Environmental regulation, Green production, Green performance, Farmers joining in cooperatives, Regulatory effect, Environmental sciences, Environmental social sciences

## Abstract

Based on 491 farmers joining in cooperatives microscopic data in Jiangxi Province,the paper uses Ordinary Least Squares to test the influence mechanism of cooperative green production on green performance, and takes environmental regulation as a regulatory variable to explore the relationship between cooperative green production and cooperative green performance. The results have shown that: (1) The green production cooperatives have a significant positive impact on their green performance, and the impact of green production on economic performance, social performance and ecological performance gradually strengthens from weak to strong; (2) Environmental regulations have a positive regulatory effect on the relationship between cooperative green production and cooperative green performance, among which three types of environmental regulations, namely, incentive, restraint and guided, can strengthen the positive relationship between green production and green performance.

## Introduction

Green is the background color of agriculture, and promoting the green development of agriculture is a profound change in the concept of agricultural development^1^. Since the Fifth Plenary Session of the 18th CPC Central Committee put forward the concept of "green development" for the first time, agricultural green development, as an important part of ecological civilization construction, has been repeatedly mentioned in important policy documents of the central government over the years. After 2018, the "No. 1 central Document" of the central government has emphasized the development of agricultural green transformation. In August 2021, the "Fourteenth Five-Year Plan" issued by six ministries and commissions, including the Ministry of Agriculture and Rural Affairs, proposed that by 2025, the green development of agriculture will be comprehensively promoted, and significant progress will be made in promoting the green transformation of rural production and lifestyle^1,2^. The successive follow-up of a series of policy measures has provided a good policy supply for the green transformation and development of agriculture.

However, it has certain limitations to promote the green development of agriculture only by the power of the government. Therefore, it is necessary to introduce rural cooperative economic organizations to make up for the limitations of government regulation. Since the promulgation of the Law on Farmers' Professional Cooperatives, Farmers' professional cooperatives have developed rapidly in China. At present, the number of registered cooperatives has exceeded 2.2 million, which has driven nearly half of the farmers and has grown into one of the most important new agricultural management mainstay in China. Undeniably, cooperatives have really played an important role in promoting the green development of agriculture by publicizing the concept of green agriculture, popularizing green agricultural technology, improving green efficiency, shaping green agricultural brands and standardizing green agricultural production, thus forming green supply^3^. However, in order to promote agricultural green production, cooperatives are faced with problems, such as asymmetric information, lack of effective supervision, and members' adverse selection, which makes it difficult to ensure the unity of agricultural product quality in green output^4^. These problems seriously restrict the farmers joining in cooperatives green development performance. Therefore, it is particularly important to explore the factors that can effectively promote the green performance of farmers joining in cooperatives.

The research on farmers joining in cooperatives the green performance in China and abroad is mainly divided into the following points. The first, how to build farmers joining in cooperatives green performance index system. Buhuabai^5^ considered that farmers joining in cooperatives the green performance can be divided into economic performance, ecological performance and social performance. The second, which the analysis factors can effect farmers joining in cooperatives green performance. It involves the personal endowment, production and operation characteristics, market environment characteristics and government regulation characteristics of cooperative members^6–10^. The third, how to categorize the types of farmers joining in cooperatives green production. Wan and Cai^11^ study on measuring green production from the adoption level of soil testing and formula fertilization technology. Zhu et al.^12^ considered that technology-intensive technology is one of the most important links in the adoption of green production technology. Guo and Wu^13^ considered that the standardized production of farmers joining in cooperatives is also the embodiment of green production. However, the academia has not explored influence on farmers joining in cooperatives green performance from the perspective of environmental regulation.

Some scholars have investigated the influence of environmental regulation on the green production behavior about farmers' cooperatives, but the research scope has not involved the green performance of farmers joining in cooperatives. From the perspective of incentive regulation, some scholars believe that the subsidy policy encourages farmers joining in cooperatives to adopt green production technology through the loss sharing mechanism^14^, but some scholars find that the subsidy policy has some problems such as narrow scope, low standard and slow speed, and the subsidy policy is an urgent to standardize the green production of cooperatives^15^. From the perspective of restraint regulation, such as, supervision and punishment are the most influential policies on cooperative green production among combined regulatory policies^16^, but some scholars have found that the imperfect government supervision system weakens the implementation effect of mandatory regulation, and cooperatives violate contracts and do not plant agricultural products according to standards^17^. From the perspective of guided regulation, policy propaganda and technical train can improve the awareness of green policies of cooperatives, which can improve their green cognition level and guide Cooperative members to implement green production^18,19^.

To sum up, previous studies focused on the influence of environmental regulation on the farmers joining in cooperatives green production behavior, but conclusion has not been consistent. Few scholars paid attention to the comprehensive evaluation of green performance by environmental regulation, let alone the in-depth analysis of the mechanism among environmental regulation, green production and green performance. In view of this, on the one hand, this paper takes farmers joining in cooperatives as the research object, and based on the "triple bottom line" theory, constructs the green performance evaluation system of farmers joining in cooperatives from the economic, ecological and social dimensions. On the other hand, environmental regulation, green production and green performance are brought into the same research framework, and environmental regulation is described from three dimensions: incentive, constraint and guided, which are used as regulatory variables to further analyze the regulatory role of environmental regulation in the impact of green production on green performance, with a view to supplementing the research on the relationship between the three.

## Theoretical analysis and research hypothesis

### Green production and green performance

Green production is a collective concept, and different production activities contain different specific green production behaviors. At present, scholars have different definitions of green production. Therefore, this paper integrates the existing research and defines agricultural green production: Green production as a mode of production that improves the added value of agriculture, saves production resources and promotes environmental pollution^20,21^. According to the specific means of green production, the green production of farmers joining in cooperatives is divided into three dimensions: green agricultural materials, green equipment and green technology, combined with the theory of "triple bottom line"^22^. It is considered that the green performance of farmers joining in cooperatives is to evaluate the economic, ecological and social performance.

Under the background of rural revitalization and green development strategy, agricultural entrepreneurial opportunities for improving environmental quality are also increasing^23^. The identification and transformation of these opportunities will certainly improve the green performance of farmers joining in cooperatives. In addition, according to the theory of ecological modernization, enterprises can obtain a win–win situation of ecological performance and economic performance through the practice of green environmental protection innovation^24^. On the one hand, green environmental protection innovation can reduce the cost of pollution control and illegal punishment of enterprises, on the other hand, it can meet the increased green consumption demand of customers by providing environmentally friendly products, improve the reputation and social benefits of enterprises, and then gain more market share and sales income. Li and Ye^25^ had tested the mediating effect of ecological performance on enterprise's green environmental innovation practice and economic performance, and concluded that enterprises can improve their ecological performance through green environmental innovation practice, and then improve their economic performance through better ecological performance.

Compared with traditional agricultural production, the cost of agricultural green production is higher. Moreover, although the green development of agriculture has obvious ecological benefits, based on the rational small-scale peasant theory, agricultural operators obtained market income in agricultural green production is the key to stimulate their production willness. Only by linking green production with green performance, cooperative members can continue to adopt green production and management methods. Cooperatives, by nature, have organizational advantages, which can better organize farmers, give play to organizational functions (unified green technology training, unified green product acquisition, unified green technology service, unified green material supply, unified green quality standards) and scale effect, reduce the input–output costs and market transaction costs of scattered farmers, make it easier to realize collective action of green production, improve agricultural green production efficiency, and achieve the transformation and improvement from green production to green performance. In the end, it will help cooperatives to improve their ecological performance and social performance by virtue of the positive externalities of green production, and effectively increase their competitive advantage and improve their economic performance through better ecological performance and social performance, thus promoting the overall improvement of their green performance. Based on this, it puts forward the following research hypotheses:

#### H1

Green production of farmers joining in cooperatives has a significant positive impact on green performance.

#### H1a

Green production of farmers joining in cooperatives has a significant positive impact on economic performance.

#### H1b

Green production of farmers joining in cooperatives has a significant positive impact on ecological performance.

#### H1c

Green production of farmers joining in cooperatives has a significant positive impact on social performance.

### The regulatory role of environmental regulation

Environmental regulation has a regulatory effect on the influence of cooperative green production on its green performance. Because the market mechanism can't completely solve the negative externality of individual behavior, government intervention can make up for the market failure and make it possible to achieve Pareto optimal resource allocation. The Organization for Economic Cooperation and Development (OECD) classifies environmental regulation into three types: economic incentive, command control and persuasion^26^. Referring to the above classification, the paper divides environmental regulation into three categories: incentive, constraint and guided. Incentive environmental regulation refers to a series of economic compensation or rewards implemented by the government to encourage producers to use environmental protection technologies^26^. Constraint environmental regulation is formulated by the legislative department or the entrusted administrative department, aiming at directly influencing the laws, regulations, policies and systems that producers make favorable environmental protection choices^27^. Guided regulation refers to the norm that government departments actively guide producers to carry out environmental protection production through propaganda, training, guidance and other agricultural technology extension activities^28^.

Neo-classical economics theory holds that the implementation of environmental regulation will increase producers' pollution control costs and related R&D investment, thus inhibiting green production and producing "following costs" benefits^29^. The Porter Hypothesis holds a negative attitude to this view, arguing that the implementation of environmental regulation can encourage producers to adopt cleaner production technology, optimize the efficiency of factor allocation, partially or even completely offset their "compliance cost", realize the dual goals of ecological effect and economic effect, and produce the "innovation compensation" effect^30^. Since Porter hypothesis was put forward, most empirical studies have supported that environmental regulation can produce incentive effect. Xu et al.^31^ concluded that environmental regulation can effectively improve people's profit-seeking behavior, and both incentive and constraint environmental regulation will play a positive regulatory role between cognition and agricultural green production willingness. In addition, according to the prospect theory, actors are more sensitive to losses than to gains. In order to avoid administrative punishment, producers are more likely to abide by relevant constraint environmental regulations and carry out green production^32^. Guided environmental regulation provides important information support and technical support for farmers joining in cooperatives, and also helps cooperatives actively implement green production to enhance their green performance. In view of this, the paper puts forward the following assumptions:

#### H2

Environmental regulation has a positive regulatory effect on the relationship between green production and green performance.

#### H2a

Incentive environmental regulation has a positive regulatory effect on the relationship between green production and green performance.

#### H2b

Constraint environmental regulation has a positive regulatory effect on the relationship between green production and green performance.

#### H2c

Guided environmental regulation has a positive regulatory effect on the relationship between green production and green performance.

**Figure 1:**
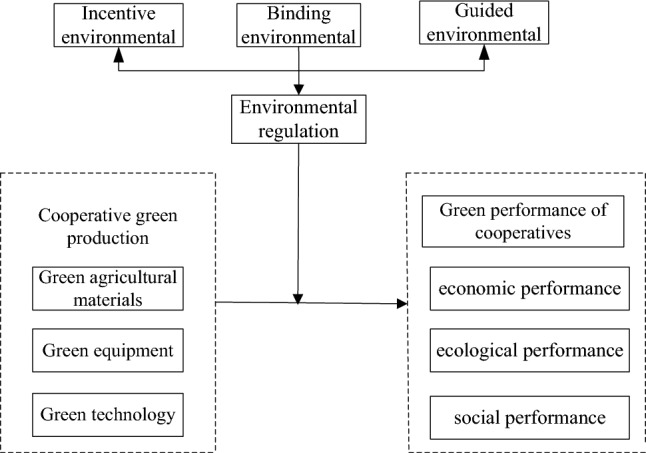
Mechanism model of cooperative green production on cooperative performance based on the regulatory effect of environmental regulation.

Based on the above analysis, the paper takes environmental regulation as the regulating variable, and constructs the influence mechanism about between cooperative green production on cooperative green performance (Fig. 1).

### Data source, variable description and model setting

#### Data sources

The data used in this paper comes from the questionnaire survey of farmers joining in cooperatives in Jiangxi Province, and the pre-survey data comes from the questionnaire survey of the directors attending the training course of new agricultural business entities in Jiangxi Province in October 2022. The formal survey questionnaires were distributed by relying on the staff of agricultural committees and agricultural economic departments in Jiangxi Province from October to November 2022, and a total of 519 questionnaires were distributed, 28 invalid questionnaires with missing content or distorted information were eliminated, and 491 valid questionnaires were finally recovered, with an effective rate of 94.61. The survey sample covers 11 districts and cities such as Nanchang City, Fuzhou City and Shangrao City in Jiangxi Province, involving 61 counties (districts) such as Nanchang County, Zixi County and Guangxin District. The sample is widely distributed in Jiangxi Province and has certain representativeness. In addition, there are obvious differences in specific green production behaviors among different types of farmers' cooperatives (planting, breeding, agricultural products processing, etc.). In order to make the research more targeted, the paper takes planting farmers' cooperatives as an example, focusing on green production and green performance of planting, and ensuring the rigorous and accurate research conclusions.

Sample areas were selected by stratification (prefecture-level city/county) and random sampling of towns (townships) and villages, and stratified sampling was used to investigate the green production and green performance samples of farmers joining cooperative planting. The farmers joining in cooperatives in Jiangxi Province were selected as the sample for two reasons: First, the farmers joining in cooperatives in Jiangxi Province developed rapidly from 19,100 in 2012 to 79,000 in 2023; Second, the number of national, provincial, municipal and county-level peasant cooperative demonstration cooperatives in Jiangxi Province has reached 468, 1184, more than 2200 and 3500, respectively, and the proportion of cooperative demonstration cooperatives has further increased.

#### Description of variables

The variable explained in this paper is green performance, which can be divided into economic performance^33^, ecological performance^34^ and social performance. The explanatory variables are green production, including green agricultural materials, green equipment and green technology^35^. The regulate variable is environmental regulation, which can be further divided into incentive type and constraint type^36^ and guide type. The control variables are capital endowment and organizational attributes, in which capital endowment is divided into five dimensions: natural capital, material capital, human capital, economic capital and social capital based on the sustainable livelihood framework^37^.

Except for organizational attributes, all the measurement items in the questionnaire use Likert5 scale. Respondents are asked to indicate to what extent they agree with the relevant measurement items, where 1–5 respectively indicate: totally disagree, comparatively disagree, generally, comparatively agree and totally agree. Finally, five groups of variables, a total of 47 items, are determined. The specific variables and descriptive statistical characteristics are as follows Table 1 shown:

**Table 1:** Variable explanation and descriptive statistical variable.

Variable	Name		Definition and assignment	Mean	SD
Be explained variable	Green performance (GP)	Economic performance (GP1)	Compared with the peers who have not carried out green production, the profit (surplus) of our products is higher (GP1–1)	3.976	0.925
			Compared with the peers who have not carried out green production, our cooperative has higher asset utilization efficiency (GP1–2)	4.026	0.865
			Compared with peers who have not carried out green production, our products sell better (GP1–3)	4.071	0.867
		Ecological performance (GP2)	Compared with the peers who have not carried out green production, our cooperative consumes less resources (GP2–1)	3.988	0.926
			Compared with the peers who have not carried out green production, our cooperative produces less pollution (GP2–2)	4.179	0.841
			Compared with the peers who have not carried out green production, the recycling rate of waste in our cooperative is higher (GP2–3)	4.173	0.821
		Social performance (GP3)	Compared with the peers who have not carried out green production, our cooperative has improved the cognition and quality of local villagers (GP3–1)	4.191	0.806
			Compared with the peers who have not carried out green production, the income of our members has increased faster (GP3–2)	4.081	0.851
			Compared with the peers who have not carried out green production, our cooperative has improved the local rural governance capacity (GP3–3)	4.193	0.806
Explanatory variable	Green production (GB)	Green agricultural materials (GB1)	Green pesticide (GB1–1) will be used when applying pesticide	4.430	0.756
			Organic fertilizer (GB1–2) will be used when fertilizing	4.462	0.729
			Product selection: Green and recyclable packaging (GB1–3)	4.344	0.817
		Green equipment (GB2)	Water-saving equipment (GB2–1) will be used for irrigation	4.381	0.777
			Low energy consumption equipment (GB2–2) will be used in production	4.330	0.749
			Precision equipment (GB2–3) will be used in production	4.281	0.820
		Green technology (GB3)	Soil testing formula technology (GB3–1) will be used in fertilization	4.234	0.836
			Green technology (GB3–2) will be used in weeding and pest control	4.360	0.810
			Conservation tillage (GB3–3) is selected for soil tillage	4.385	0.764
			Waste discharge will be treated in a green way (GB3–4)	4.354	0.846
			Clean energy (GB3–5) will be selected for energy use	4.338	0.813
regulated variable	Environmental regulation (ER)	Incentive type (ER1)	Green agricultural credit subsidies promote green production of our cooperative (ER1–1);	4.291	0.859
			Green agricultural insurance premium subsidies promote green production of our cooperative (ER1–2)	4.283	0.845
			Green agriculture tax subsidies promote green production of our cooperative” (ER1–3)	4.257	0.861
			"‘Three Products and One Standard’ subsidies promote green production of our cooperative” (ER1–4)	4.316	0.826
			The subsidy of the main demonstration level promotes the green production of the cooperative (ER1–5)	4.316	0.759
			Industrial park demonstration park subsidies promote the green production of our cooperative (ER1–6)	4.216	0.854
			Subsidies for protection of cultivated land fertility to promote green production of our cooperative (ER1–7)	4.322	0.779
			Waste resource subsidies promote green production of our cooperative (ER1–8)	4.232	0.854
			Subsidies for fallow farmland rotation to promote green production in our cooperative (ER1–9)	4.244	0.834
			Comprehensive subsidies for green agricultural materials promote green production of our cooperative (ER1–10)	4.299	0.779
			Application of subsidies for agricultural machinery purchase promote green production of our cooperative (ER1–11)	4.346	0.747
			Green agricultural technology extension subsidies promote green production of our cooperative (ER1–12)	4.367	0.745
		Constrained type (ER2)	Existing production standards promote green production (ER2–1)	4.340	0.726
			Testing existing products to promote green production (ER2–2)	4.330	0.754
			Existing penalties promote green production (ER2–3)	4.204	0.864
		Guiding type (ER3)	The existing on-site guidance can meet the demand of green production (ER3–1)	4.334	0.763
			The existing lecture training can meet the demand of green production (ER3–2)	4.310	0.760
			The existing field visit can meet the demand of green production (ER3–3)	4.316	0.737
			The existing publicity materials can meet the demand of green production (ER3–4)	4.255	0.789
Control variable	Capital endowment	Natural capital	The quality of the land operated by this cooperative is good (Nat)	4.118	0.817
		physical capital	The fluctuation of our annual income is relatively stable (Sub)	3.923	0.907
		manpower capital	Our members are more supportive of green production (Lab)	4.348	0.681
		Economic capital	It is easier for our agency to obtain bank loans (Eco)	3.847	0.997
		social capita	Our cooperative has easy access to green production technology (Soc)	4.238	0.752
	Organizational attribute	Leading subject	The cooperative's start-up mode (Pat) is: capable person-led, leading enterprise-led, rural service department-initiated, government-initiated, supply and marketing cooperatives-led and village cadres-led: government-initiated or village cadres-led = 1; Other = 0	0.134	0.341
		Demonstration grade	The cooperative's demonstration grade (Gra) is: Non-model society = 1; County-level demonstration society = 2; Municipal Model Society = 3; Provincial Model Society = 4; National Model Society = 5	2.415	1.359
		management mode	Our organization and management include (Adm) unified technical training, unified product acquisition, unified technical service, unified material supply, and unified quality standards: one type is adopted = 1; Adopt two kinds = 2; Adopt three kinds = 3; Adopt 4 kinds = 4; Adopt 5 kinds = 5	2.629	1.528

Because the subsequent empirical part intends to use OLS ordinary least square method for analysis, and in consideration of the questionnaire design, the variables of green performance, green production and environmental regulation are characterized by several items by building an index system. Therefore, according to the factor analysis method and the factor load, the weight of each index is calculated, and the comprehensive index of green performance, green production and environmental regulation is obtained. See the specific weight in Table 2.

**Table 2:** Weight assignment table.

Target layer	Primary index	Observation index	Weight
Green performance (GP)	Economic performance (GP1)	Compared with the peers who have not carried out green production, the profit (surplus) of our products is higher (GP1–1)	0.106
		Compared with the peers who have not carried out green production, our cooperative has higher asset utilization efficiency (GP1–2)	0.114
		Compared with peers who have not carried out green production, our products sell better (GP1-3)	0.112
	Ecological performance (GP2)	Compared with the peers who have not carried out green production, our cooperative consumes less resources (GP2–1)	0.108
		Compared with the peers who have not carried out green production, our cooperative produces less pollution (GP2–2)	0.108
		Compared with the peers who have not carried out green production, the recycling rate of waste in our cooperative is higher (GP2–3)	0.111
	Social performance (GP3)	Compared with the peers who have not carried out green production, our cooperative has improved the cognition and quality of local villagers (GP3–1)	0.113
		Compared with the peers who have not carried out green production, the income of our members has increased faster (GP3–2)	0.114
		Compared with the peers who have not carried out green production, our cooperative has improved the local rural governance capacity (GP3–3)	0.113
Green production (GB)	Green agricultural materials (GB1)	Green pesticide (GB1–1) will be used when applying pesticide	0.088
		Organic fertilizer (GB1–2) will be used when fertilizing	0.091
		Product selection: Green and recyclable packaging (GB1–3)	0.089
	Green equipment (GB2)	Water-saving equipment (GB2–1) will be used for irrigation	0.092
		Low energy consumption equipment (GB2–2) will be used in production	0.088
		Precision equipment (GB2–3) will be used in production	0.090
	Green technology (GB3)	Soil testing formula technology (GB3–1) will be used in fertilization	0.087
		Green technology (GB3–2) will be used in weeding and pest control	0.096
		Conservation tillage (GB3–3) is selected for soil tillage	0.097
		Waste discharge will be treated in a green way (GB3–4)	0.092
		Clean energy (GB3–5) will be selected for energy use	0.088
Environmental regulation (ER)	Incentive type (ER1)	Green agricultural credit subsidies promote green production of our cooperative (ER1–1)	0.051
		Green agricultural insurance premium subsidies promote green production of our cooperative (ER1–2)	0.052
		Green agriculture tax subsidies promote green production of our cooperative (ER1–3)	0.052
		Three products and one standard’ subsidies promote green production of our cooperative (ER1–4)	0.052
		The subsidy of the main demonstration level promotes the green production of the cooperative (ER1–5)	0.056
		Industrial park demonstration park subsidies promote the green production of our cooperative (ER1–6)	0.052
		Subsidies for protection of cultivated land fertility to promote green production of our cooperative (ER1–7)	0.054
		Waste resource subsidies promote green production of our cooperative (ER1–8)	0.052
		Subsidies for fallow farmland rotation to promote green production in our cooperative (ER1–9)	0.051
		Comprehensive subsidies for green agricultural materials to promote green production in our cooperative (ER1–10)	0.055
		Application of subsidies for agricultural machinery purchase promote green production of our cooperative (ER1–11)	0.053
		Green agricultural technology extension subsidies promote green production of our cooperative (ER1–12)	0.054
	Constrained type (ER2)	Existing production standards promote green production (ER2–1)	0.053
		Testing existing products to promote green production (ER2–2)	0.051
		Existing penalties promote green production (ER2–3)	0.051
	Guiding type (ER3)	The existing on-site guidance can meet the demand of green production (ER3–1)	0.055
		The existing lecture training can meet the demand of green production (ER3–2)	0.054
		The existing field visit can meet the demand of green production (ER3–3)	0.053
		The existing publicity materials can meet the demand of green production (ER3–4)	0.051

In addition, Cronbach's Alpha coefficient method is used to analyze the reliability of the questionnaire data, and KMO sampling appropriateness quantity, Bartlett sphericity test and aggregate validity analysis are used to demonstrate the reliability of the data validity. Cronbach's Alpha coefficients of four groups of variables except organizational attributes (non-scale questions) are respectively 0.974, 0.958, 0.978 and 0.881 (due to space limitations, the test results are not listed and kept for reference), and the overall Cronbach's Alpha coefficient reaches 0.981, which is higher than the criterion of 0.6, so it can be considered that the reliability level of questionnaire data is good. The KMO values of the four groups of variables are all greater than the threshold condition of 0.5, and the Bartlett sphericity test is significant at the statistical level of 1%. The overall KMO measurement value of the sampling adequacy is 0.969, and the approximate chi-square value of the Bartlett sphericity test is 26,119.914(df = 946, sig. < 0.001), which reflects that the questionnaire data is suitable for factor analysis. The confirmatory analysis of factor load is carried out by AMOS 26.0, and it is concluded that the load coefficients of the four groups of variables on the observed variables are all higher than the threshold condition of 0.6, which shows that the questionnaire data has sufficient validity. To sum up, the questionnaire data is reliable and valid enough for further data analysis.

#### Model setting

This paper adopts Ordinary Least Squares. In order to verify H1: the green production of farmers' cooperatives has a significant positive impact on green performance, and the sub-hypotheses H1a, H1b and H1c, the following model is constructed:

1 $$ \begin{gathered} {GP_{i}} = {\beta_{0}} + {\beta_{1}} GB + {\beta_{2}} Nat + {\beta_{3}} Sub + {\beta_{4}} Lab + {\beta_{5}} Eco + {\beta_{6}} Soc + {\beta_{7}} Pat + {\beta_{8}} Gra + {\beta_{9}} Adm + \varepsilon \hfill \end{gathered} $$

2 $$ \begin{gathered} GP_{i} = \beta_{0} + \beta_{1} GB + \beta_{2} Nat + \beta_{3} Sub + \beta_{4} Lab + \beta_{5} Eco + \beta_{6} Soc + \beta_{7} Pat + \beta_{8} Gra + \beta_{9} Adm + \varepsilon \hfill \\ (i = 1,2,3) \hfill \\ \end{gathered} $$

From the formula (2), GP_i_ (i = 1,2,3) stands for three kinds of green production performance

3 $${\text{GP}}_{\text{i}}={\upgamma }_{0}+{\upgamma }_{1}\text{GB}\times \text{ER}+{\upgamma }_{2}\text{Nat}+{\upgamma }_{3}\text{Sub}+{\upgamma }_{4}\text{Lab}+{\upgamma }_{5}\text{Eco}+{\upgamma }_{6}\text{Soc}+{\upgamma }_{7}\text{Pat}+{\upgamma }_{8}\text{Gra}+{\upgamma }_{9}\text{Adm}+\upsigma $$

From the formula (3), in order to verify that H2: environmental regulation has a positive regulatory effect on the relationship between green production and green performance, and the sub-hypotheses H2a, H2b and H2c, and to eliminate the collinearity of the sum of variables and the sum of interactive items, the sum of variables is "centered" to obtain the sum of variables, and the interactive items are introduced and regressed in the above model and interactive item ER and Collinearity problem, GB and carry out centering processing carry out regression.

### Empirical results and analysis

#### Correlation analysis

In order to explore the correlation between variables and verify whether there are multiple collinearity problems, Pearson correlation coefficient test and VIF variance expansion factor test are carried out before formal regression (Table 3).

**Table 3:** Pearson correlation coefficient test table.

	GP	GB	Nat	Sub	Lab	Eco	Soc	Pat	Gra	Adm
GP	1									
GB	0.677***	1								
Nat	0.611***	0.531***	1							
Sub	0.647***	0.532***	0.756***	1						
Lab	0.632***	0.627***	0.589***	0.569***	1					
Eco	0.582***	0.479***	0.583***	0.661***	0.499***	1				
Soc	0.627***	0.609***	0.605***	0.596***	0.726***	0.525***	1			
Pat	− 0.021	− 0.044	0.031	0.034	− 0.061	0.006	0.002	1		
Gra	0.160***	0.147***	0.06	0.061	0.137***	− 0.036	0.129***	− 0.156***	1	
Adm	0.106**	0.107**	0.017	− 0.012	0.081*	− 0.043	0.052	− 0.112**	0.286***	1

By Table 4. It can be seen that there is a significant positive correlation between green production (GB) and green performance (GP), which is consistent with the assumption and expectation of H1, and the correlation coefficients between variables are basically below the threshold of 0.8. In addition, the VIF test results show that the variance expansion factor is basically lower than the empirical value of 3 and less than 10, which indicates that there is no multicollinearity problem and the next regression study can be carried out.

**Table 4:** VIF variance expansion factor test table.

Variable	GB	Nat	Sub	Lab	Eco	Soc	Pat	Gra	Adm
VIF	1.96	2.67	2.95	2.54	1.96	2.56	1.04	1.15	1.11

#### Basic model analysis

In addition to discussing the influence of green production on green performance, this paper also analyzes the role of green production on each specific performance under green performance, in order to clarify how green production mainly improves the green performance level of farmers' cooperatives.

Table 5 can be seen that the influence coefficient of green production on green performance is 0.364, which is significant at the level of 1%. The H1 hypothesis proves that green production of farmers' cooperatives has a significant positive impact on green performance. At the same time, the influence coefficient of green production on economic performance, ecological performance and social performance is positive, and it is significant at the level of 1%. The sub-hypotheses H1a, H1b and H1c of H1 are also confirmed.

**Table 5:** Regression analysis results in the influence of green production on green performance.

Variable	Green performance (GP)	Green performance_economic performance (GP1)	Green performance_ecological performance (GP2)	Green performance_social performance (GP3)
Green production (GB)	0.364*** (8.01)	0.310*** (6.00)	0.404*** (8.31)	0.380*** (7.79)
Natural capital (Nat)	0.072* (1.65)	− 0.018 (− 0.36)	0.154*** (3.30)	0.082* (1.76)
Material capital (Sub)	0.163*** (3.96)	0.292*** (6.22)	0.089** (2.01)	0.108** (2.44)
Human capital (Lab)	0.144*** (2.82)	0.133** (2.30)	0.078 (1.44)	0.219*** (4.01)
Economic capital (Eco)	0.121*** (3.95)	0.154*** (4.42)	0.118*** (3.60)	0.089*** (2.72)
Social capital (Soc)	0.100** (2.16)	0.104** (1.98)	0.111** (2.24)	0.085* (1.70)
Leading entity (Pat)	0.013 (0.20)	0.006 (0.07)	0.024 (0.35)	0.012 (0.17)
Demonstration grade (Gra)	0.035** (2.03)	0.038* (1.95)	0.038** (2.06)	0.029 (1.57)
Management mode (Adm)	0.025* (1.66)	0.026 (1.50)	0.020 (1.23)	0.029* (1.83)
Constant term	− 0.090 (− 0.55)	− 0.168 (− 0.91)	− 0.040 (− 0.23)	− 0.063 (− 0.36)
Observed value	491	491	491	491
$R^{2}$	0.624	0.590	0.584	0.579
Adjustment $R^{2}$	0.617	0.582	0.576	0.571

***p < 0.01, **p < 0.05, *p < 0.1, the values in brackets are t values, the same below.

In addition, from the coefficient, we can see that the influence of green production on economic performance, social performance and ecological performance gradually strengthens from weak to strong, which shows that green production can mainly improve the social performance and ecological performance of farmers' cooperatives, which is consistent with the positive externalities of green production, while the cost of green production for farmers joining in cooperatives is higher than that of traditional production methods, and the contribution of green production to promoting economic performance is relatively small.

In practice, this result is also easy to understand. First of all, in terms of improving social performance, cooperatives, as an important production, operation and service subject, can promote small farmers to carry out green production by establishing a stable interest linkage mechanism and providing necessary public services, so as to promote the growth of their members' income, and by virtue of their demonstration and leading role in the village, enhance the villagers' green cognition and literacy, thus enhancing the rural governance capacity and bringing about the improvement of the social performance of cooperatives. Secondly, in order to improve ecological performance, the implementation of green production by cooperatives can reduce the discharge of waste, wastewater and waste gas and the occurrence of environmental accidents, which is conducive to improving the rural ecological environment, establishing the environmental image of cooperatives and improving their ecological performance. Finally, in improving economic performance, green production has a large investment in the early stage, and there may be a lag effect, so it is difficult to achieve economic performance in the short term. Therefore, green production needs to be further strengthened in promoting economic performance, especially in improving farmer joining in cooperatives.

At the same time, the binary variable of environmental regulation (low environmental regulation: environmental regulation score < 4 = 0; High environmental regulation: the score of environmental regulation is ≥ 4 = 1), and the samples of farmers joining in cooperatives under the influence of low environmental regulation and high environmental regulation are regressed in groups. By comparing the influence coefficient and significance level of green production on green performance, it is preliminarily judged whether environmental regulation has a regulate effect on the influence degree between green production and green performance.

According to Table 6. It can be seen that whether under the influence of low environmental regulation or high environmental regulation, green production positively affects green performance at a significant level of 1%, but the green production coefficient is higher in the high environmental regulation group, which indicates that environmental regulation will amplify the influence of green production and green performance, which can be analyzed in detail through the next step of environmental regulation adjustment effect test.

**Table 6:** Regression analysis table of the influence of green production on green performance under different environmental regulations.

Variable	Green performance (GP)	Under the influence of low environmental regulation	Under the influence of high environmental regulations
		Green performance (GP)	Green performance (GP)
Green production (GB)	0.364*** (8.01)	0.279*** (4.16)	0.450*** (7.49)
Natural capital (Nat)	0.072* (1.65)	0.134* (1.97)	0.033 (0.60)
Material capital (Sub)	0.163*** (3.96)	0.102 (1.38)	0.164*** (3.22)
Human capital (Lab)	0.144*** (2.82)	0.184** (2.41)	0.146** (2.10)
Economic capital (Eco)	0.121*** (3.95)	0.029 (0.55)	0.157*** (4.17)
Social capital (Soc)	0.100** (2.16)	0.140* (1.85)	0.088 (1.50)
Leading entity (Pat)	0.013 (0.20)	− 0.077 (− 0.71)	0.056 (0.70)
Demonstration grade (Gra)	0.035** (2.03)	0.033 (1.01)	0.034* (1.69)
Management mode (Adm)	0.025* (1.66)	− 0.002 (− 0.06)	0.037** (2.06)
Constant term	− 0.090 (− 0.55)	0.330 (1.15)	− 0.457* (− 1.72)
Observed value	491	128	363
$R^{2}$	0.624	0.537	0.560
Adjustment $R^{2}$	0.617	0.502	0.548

#### Regulatory effect test

Green production, overall environmental regulation and three different types of environmental regulation and their interactions after "centralization" are put into the model for regression, and the results are as follows. Table 7 as shown. The direction of the cross-product coefficient and the direction of the main effect coefficient are consistent, and which are significant at the level of 1%, indicating that both the overall environmental regulation and the three types of environmental regulation strengthen the relationship between green production and green performance, and H2 and its sub-hypotheses H2a, H2b and H2c are verified. Incentive, restrictive, and guided environmental regulations can all be used as effective means. They can promote the green production of farmers joining cooperatives and improve green performance.

**Table 7:** Regulatory effect test table.

Variable	Green performance (GP)			
Green production ($GB^{\# }$)	0.649*** (12.98)	0.664*** (13.72)	0.640*** (13.45)	0.700*** (14.29)
Environmental regulation ($ER^{\# }$)	0.297*** (6.38)			
Environmental regulation_incentive ($ER_{1}^{\# }$)		0.275*** (6.29)		
Environmental regulation_constrained ($ER_{2}^{\# }$)			0.290*** (6.80)	
Environmental regulation_guided ($GB^{\# }$)				0.219*** (4.97)
Green production × environmental regulation ($GB^{\# } \times ER_{{}}^{\# }$)	0.154*** (3.02)			
Green production × environmental regulation_incentive ($GB^{\# } \times ER_{1}^{\# }$)		0.151*** (3.15)		
Green production × environmental regulation_constrained ($GB^{\# } \times ER_{2}^{\# }$)			0.149*** (2.98)	
Green production × environmental regulation_guided ($GB^{\# } \times ER_{3}^{\# }$)				0.160*** (3.28)
Observed value	491	491	491	491
$R^{2}$	0.513	0.511	0.514	0.497
After adjustment $R^{2}$	0.510	0.508	0.511	0.494

Incentive environmental regulation can provide effective resources and market support for cooperative green production, reduce the use cost of cooperative green agricultural materials, green equipment and green technology, and improve the degree of cooperative green input into green performance. Although constraint environmental regulation may increase the pollution control cost of cooperatives in the short term, cause unproductive investment and crowd out productive investment, in the long run, the positive benefits of "innovation compensation" will offset or even surpass the negative effects of "following costs". Moreover, there are basically no governance costs and high administrative penalties for cooperatives that abide by binding environmental regulations, so it is easy for cooperatives under constraint environmental regulations to improve their green performance through green production. In addition, the guiding guided environmental regulation provides an effective external link for cooperatives, which helps cooperatives to carry out green production more systematically and standardized, improve their agricultural production efficiency, and then realize the promotion of green performance.

### Robustness test

#### Change the measurement method of main variables

In this paper, factor analysis is used to calculate the comprehensive index of green production and green performance in empirical analysis. In order to ensure that the empirical results are not established by chance because of the selective use of specific measurement methods, the measurement methods of main variables are replaced by the average method, and the green production (GB_), green performance (GP_), economic performance (GP1_), ecological performance (GP2_) and social performance (GP3_) calculated by the average method are obtained.

From Table 8 can be seen that the research conclusion is consistent with the above, and the influence of green production on green performance and its sub-dimension performance is significant at the level of 1%, which shows that the original theoretical derivation and model construction are reasonable and will not change due to the change of measurement methods of main variables, and the empirical results are stable and reliable.

**Table 8:** Replace the main variable robustness.

Variable	Green performance (GP_)	Green performance_economic performance (GP1_)	Green performance_ecological performance (GP2_)	Green performance_social performance (GP3_)
Green production (GB_)	0.365*** (8.02)	0.311*** (6.01)	0.404*** (8.27)	0.380*** (7.80)
Natural capital (Nat)	0.073* (1.67)	− 0.018 (− 0.36)	0.153*** (3.28)	0.082* (1.76)
Material capital (Sub)	0.164*** (3.97)	0.292*** (6.22)	0.091** (2.06)	0.108** (2.45)
Human capital (Lab)	0.143*** (2.80)	0.133** (2.30)	0.076 (1.39)	0.219*** (4.01)
Economic capital (Eco)	0.121*** (3.95)	0.153*** (4.41)	0.120*** (3.64)	0.089*** (2.72)
Social capital (Soc)	0.100** (2.15)	0.104** (1.97)	0.112** (2.24)	0.084* (1.70)
Leading entity (Pat)	0.013 (0.21)	0.006 (0.08)	0.023 (0.33)	0.012 (0.17)
Demonstration grade (Gra)	0.035** (2.02)	0.038* (1.95)	0.037** (2.03)	0.029 (1.57)
Management mode (Adm)	0.025* (1.65)	0.026 (1.50)	0.020 (1.22)	0.029* (1.83)
Constant term	− 0.092 (− 0.57)	− 0.171 (− 0.93)	− 0.040 (− 0.23)	− 0.065 (− 0.37)
Observed value	491	491	491	491
$R^{2}$	0.625	0.590	0.584	0.579
After adjustment $R^{2}$	0.618	0.582	0.576	0.571

#### Propensity score matching (PSM)

The increase of the comprehensive index of green performance in the study may be due to the capital endowment and organizational attributes of farmers' cooperatives such as natural capital and economic capital. In order to solve the problem of sample self-selection, a binary variable of green production (low level green production: green production score < 4 = 0; High-level green production: the score of green production is ≥ 4 = 1). Farmers joining in cooperatives with high-level green production are taken as the treatment group, and natural capital (Nat), material capital (Sub), human capital (Lab), economic capital (Eco), social capital (Soc), leader (Pat), demonstration level (Gra) and management mode (Adm) are taken as the treatment group.

**Figure 2:**
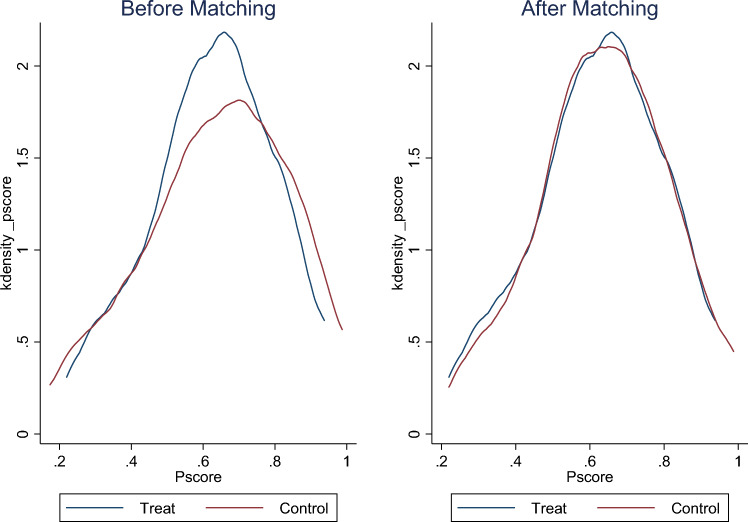
Nuclear density map before and after PSM.

From Fig. 2. It can be seen that there are many differences between the samples before matching, and after matching, except for the difference in green production, other differences have been controlled accordingly.

PSM samples the results are as follows Table 9. The coefficient of green production (GB) to green performance (GP) is 0.247, which is significant at the level of 1%, indicating that green production (GB) still has a significant positive impact on green performance (GP). However, the influence of green production on economic performance, social performance and ecological performance in green performance is from weak to strong, and the path of green production affecting green performance is mainly realized by improving social performance and ecological performance, which is mutually confirmed by the previous research, and the conclusion is still stable.

**Table 9:** PSM matched sample inspection results table.

Variable	Green performance (GP)	Green performance_economic performance (GP1)	Green performance_ecological performance (GP2)	Green performance_social performance (GP3)
Green production (GB)	0.247*** (3.78)	0.203** (2.60)	0.274*** (3.84)	0.266*** (3.71)
Natural capital (Nat)	0.092 (1.37)	0.013 (0.16)	0.125* (1.70)	0.141* (1.91)
Material capital (Sub)	0.159** (2.25)	0.320*** (3.78)	0.118 (1.52)	0.039 (0.50)
Human capital (Lab)	0.126* (1.71)	0.078 (0.89)	0.098 (1.22)	0.201** (2.48)
Economic capital (Eco)	0.049 (0.93)	0.113* (1.80)	0.053 (0.93)	− 0.019 (− 0.34)
Social capital (Soc)	0.153** (2.21)	0.129 (1.55)	0.158** (2.08)	0.172** (2.25)
Leading entity (Pat)	− 0.136 (− 1.22)	− 0.087 (− 0.65)	− 0.131 (− 1.07)	− 0.189 (− 1.54)
Demonstration grade (Gra)	0.001 (0.03)	0.017 (0.42)	0.015 (0.42)	− 0.028 (− 0.76)
Management mode (Adm)	0.091*** (3.04)	0.107*** (3.00)	0.082** (2.52)	0.082** (2.50)
Constant term	0.342 (0.81)	0.142 (0.28)	0.343 (0.74)	0.521 (1.12)
Observed value	155	155	155	155
$R^{2}$	0.355	0.317	0.317	0.317
After adjustment $R^{2}$	0.315	0.274	0.274	0.274

## Discussion

The green production motive of cooperatives arises from the multiple coupling of government environmental regulation, market demand and the development interests of cooperatives themselves. Cooperative green production may be the result of a series of organizational control and government support factors, but the core is the cooperative green development goal formed by the hard constraint of cooperative organization control and the soft incentive supported by the government.

Under the influence of high environmental regulations, the green production of farmers joining in cooperatives has improved the green performance more obviously. It shows that the government's environmental regulation, which is different from the market mechanism, has played an irreplaceable positive role in guiding farmers joining in cooperatives to participate in green production and realizing the effective conversion from green production input to green performance output, and environmental regulation has a positive regulatory effect on the relationship between green production and green performance. Moreover, three types of environmental regulations, namely incentive, constraint and guided, can strengthen the positive relationship between green production and green performance. All kinds of current environmental regulation measures in Jiangxi are conducive to promoting the implementation of green production behavior of farmers who join in cooperatives to a certain extent, and to the realization of green performance improvement.

Improve the contract governance relationship. On the basis of the existing "public–private" cooperation, the government should cultivate and give play to the restraint and drive role of grass-roots autonomous organizations such as village committees, new business entities such as cooperatives and social organizations such as industry associations in terms of policy support, business guidance and financial security, and cultivate and make them become an important participating force in promoting the transformation of green production.

## Conclusions and policy implications

Taking 491 farmers' cooperatives in Jiangxi Province as samples, this paper constructs the measurement indicators of green production, green performance and environmental regulation of farmers joining in cooperatives and measures their comprehensive index by factor analysis. It empirically tests the influencing factors of farmers joining in cooperatives' green performance by using Ordinary Least Squares, and further explores the regulatory role of environmental regulation in the relationship between green production and green performance.

Green production of farmers joining in cooperatives is helpful to improve their green performance level. Among them, green production has the strongest influence on the ecological performance under the comprehensive system of green performance, and the weakest promotion effect on the economic performance (economic performance < social performance < ecological performance). It shows that farmers joining in cooperatives have not yet exerted the maximum efficiency of green production, and need to be further strengthened in promoting the realization of the value of green production.

From this, this paper put forward the following policy enlightenment: first of all, enhance the initiative of green production of farmers joining in cooperatives. Strengthen the publicity and education of green production knowledge, improve farmers' awareness of green production, promote farmers joining in cooperatives to form a correct ecological concept and development concept, improve farmers' sense of responsibility and mission, and make them realize that green production is conducive to improving green performance and promoting the high-quality development of farmers joining in cooperatives, thus promoting the early realization of "double carbon" goals and rural ecological revitalization goals. Second, improve the realization mechanism of green production economic performance. On the one hand, from the production side, we should reasonably price green agricultural materials and green equipment, reduce the operating cost of green technology, and provide appropriate cost guarantee for the green production of farmers joining in cooperatives. On the other hand, starting from the market, we should establish an interest distribution mechanism with farmers joining in cooperatives and other agricultural producers as the main body, so that they can derive the value-added benefits of green agricultural products to a greater extent, improve the economic performance of cooperatives, and stimulate their sustainable endogenous motivation for green production. Third, optimize the regulation of green production environment of farmers joining in cooperatives. Continue to use a variety of policy tools to promote the green performance of cooperatives. First of all, we will continue to improve the incentive environmental regulations and consolidate the incentive system of green production subsidies with financial tax subsidies, demonstration incentive subsidies, environmental protection subsidies and production support subsidies as the main body. Secondly, the constraint environmental regulations should be implemented reasonably, and the green production of farmers joining in cooperatives should be moderately restrained according to local conditions, so as to formulate and implement a practical and reasonable punishment mechanism for the green production of cooperatives. Finally, the guiding environmental regulation should be strengthened. Relevant policy departments can establish a long-term education mechanism to guide the green production of farmers joining in cooperatives, strengthen relevant environmental training, and organize observation of relevant demonstration units.

## Data Availability

The datasets used and/or analysed during the current study are available from corresponding author on reasonable request.
